# The effects of received grandmothers’ affection on adult grandchildren’s health behaviors using affection exchange theory

**DOI:** 10.1186/s12889-022-13049-4

**Published:** 2022-04-11

**Authors:** Leslie Ramos Salazar, Priyanka Khandelwal, Yvette Castillo

**Affiliations:** 1 Department of Computer Information and Decision Management, West Texas A&M University, Paul & Virginia Engler College of Business, Box 60768, Canyon, TX 79016 USA; 2 Department of Marketing, University of Nebraska-Lincoln, College of Business, HLH 335P, Lincoln, NE 68588 USA; 3 Department of Education, West Texas A&M University, College of Education and Social Sciences, Box 60768, Canyon, TX 79016 USA

**Keywords:** Grandchildren, Grandparent-grandchild communication, Affection exchange theory, Affection, Health, Health behaviors

## Abstract

**Background:**

Affection exchange theory (AET) explains the value of received affection for overall wellbeing in family relationships. However, this study extends prior work by investigating AET in grandmother-grandchild relationships and grandchildren’s individual well-being. This study seeks to understand the relationships between adult grandchildren’s received grandmother affection and health-related behaviors such as diet, exercise, substance abuse, and sleep.

**Methods:**

This cross-sectional study included 229 university student participants. Multiple regression analyses were performed to analyze received grandmother affection and grandchildren’s health behaviors.

**Results:**

Using cross-sectional survey methods, it was found that grandchildren’s reports of received memories and humor, and celebratory affection influenced grandchildren’s dietary behaviors. Received love and esteem, memories and humor, and celebratory affection was also associated with grandchildren’s exercise behaviors.

**Conclusions:**

Grandchildren who receive grandmother affection may be likely to engage in the well-being process by engaging in health behaviors, while those who are not receiving affection might suffer the health consequences in adulthood. These findings support the assumption of affection exchange theory that received family affection, in this case, grandmother affection leads to positive health outcomes such as enhanced dietary and exercise behaviors among grandchildren.

**Supplementary Information:**

The online version contains supplementary material available at 10.1186/s12889-022-13049-4.

## Background

Grandparents often play a significant role in individuals’ lives. Several young adults report that their relationship with their grandparents is one of their most valued relationships [1, 2]. Young adults report receiving much of their early caregiving from one, or two grandparents [3]. A Pew Research Center report from 2013 indicated that 7 million grandparents served as their grandchildren’s caregivers and 7.7 million grandchildren were living with their grandparents, and 3 million of these grandchildren had a grandparent as their primary caregiver who fulfilled the grandchildren’s basic needs (e.g., food; shelter), which was a 22% increase from 2000, and 22% of grandparents provided regular care [4, 5]. Early parenthood, divorce, and separation from parents can sometimes transfer the caregiving role responsibility to the grandparents [4, 6]. Not only do grandparents provide basic resources to fulfill their basic needs (e.g., food; shelter), but they also nurture the well-being of their grandchildren by providing affection to them [7]. Grandparents’ affection has been shown to have a significant positive impact in their grandchildren’s personal lives [7, 8]. A study found that grandparents report spending about 11 to 20 h a week caring and bonding with their grandchildren [9].

Grandparent-grandchild communication can also play a role in grandchildren’s individual well-being. For instance, research supports the idea that withholding affection from a young adult can have negative health effects such as stress and mental health issues (i.e., depression) [10, 11], but some evidence suggests that received grandparent affection is not significantly inversely related with grandchildren’s health outcomes (i.e., stress) [12]. However, evidence suggests that when grandmother communicate affectionately to their grandchildren, the expressed affection strengthens the grandmother-grandchild relationship and it enhances grandmothers’ general well-being [13]. However, while researchers have investigated grandchildren’s perspectives on their relationship with their grandmother, the construct of grandmother received affection has been understudied in relation to grandchildren’s health outcomes. It is important to examine the positive influence of received grandmother affection on young adults’ health behaviors because these relationships can inform about the value of intergenerational relationships from a health perspective. Because received affection has been shown to be beneficial to the health of other family relationships such as parental and romantic [14], this study will aim at investigating the grandmother-grandchild relationship using Affection Exchange Theory, which states that received affection may lead to positive health outcomes [15]. As such, the objective of this paper is to examine the role of perceived grandparents’ affection and their adult grandchildren’s perceived health behavioral outcomes.

### Grandparent-grandchild relationships

The grandparent-grandchild (GP-GC) relationship is one of the most esteemed relationships that a grandchild can have [16]. Studies have shown that grandchildren tend to view their grandparent relationship almost as important as their relationship with their parental relationship [17, 18]. Because college-aged grandchildren value their relationships with their grandparents they tend to use behaviors to maintain their grandparent-grandchild relationship [19, 20]. For instance, grandchildren report engaging in relationship maintenance behaviors, or actions such as being more optimistic in interactions, the use of conflict management, using assurances, and openness to maintain their relationship with their grandparent [19]. These relational maintenance behaviors ensure that a quality GP-GC relationship is maintained [21].

It has also been shown that college student grandchildren are driven to communicate regularly with their grandparents [19]. For instance, when college students are in desperation, or in moments of distress, they tend to increase their communication with their grandparents to provide financial or emotional support [22]. Rarely, it is the case that grandparents live in the same household as the grandchild, but in some culturally diverse families (e.g., Latino families, Asian-American families) adult grandchildren report living with their grandparents, which enhance the frequency of their everyday communication [23, 24]. However, the average grandparent-grandchild relationship of students attending college live away from their grandparents and this (GP-GC) relationship is sustained as a long-distance relationship [19]. These long-distance GP-GC relationships are maintained by using a variety of communication channels such as face-to-face, telephone, and email [25].

Researchers have found several factors that influence GP-GC interactions including perceived relational closeness [26], accommodating interactive patterns [27], self-disclosure [28], and conversation topics [29]. Through the communication process grandchildren tend to learn values, attitudes toward aging, and family histories [30, 20]. The sex of the grandparent also has an effect in shaping the range of topics that are discussed during a conversation. For example, in a content analysis, Nussbaum and Bettini (1994) discovered that grandmothers spoke about family matters and with greater length, while grandfathers talked about their own youth, morals, and health.

While both grandparents are valued by their grandchildren, grandmother-grandchild relationships are reported to be the closest [31]. Part of the reason is that the grandmother-grandchild relationship that is nurtured during childhood has been shown to be a positive predictor of the quality of the grandmother-grandchild relationship in adulthood [32, 33]. Another reason is that due to gender norms established by society, grandmothers are often expected to serve as nurturers and contribute to the caregiving of the early years of their grandchildren [34]. The relationship development between grandmothers and their grandchildren has been shown to be facilitated by the emotional attachment and feelings of relational closeness that have been established [35]. Grandchildren also report seeking more social and emotional support from their grandmothers than their grandfathers [36]. When encountering problems, college-age adults tend to self-disclose their problems to their grandmothers to be able to vent with someone they feel comfortable with [37]. For this reason, this investigation will focus on examining grandmother-grandchild relationships.

### Affection exchange theory and grandparent-grandchild relationships

Affection Exchange Theory (AET) is grounded in Darwin’s principle that humans exchange affection to achieve the *survival* and *procreation* of the human species [38, 39]. AET describes that humans are internally driven to both provide and receive affection in their relationships to sustain genetic fitness [39]. Affectionate communication is “defined as encompassing those behaviors that encode feelings of fondness and intense positive regard and are generally decoded as such by their intended receivers” [40] (p. 312). Affection can be expressed using verbal and nonverbal communication [38, 39]. Affection exchange theory (AET) has served as an important theory in understanding grandparent-grandchild relationships. For instance, grandparents can communicate affection to enact the liking process of their grandchildren, which can in turn, increase the likelihood that grandchildren will reciprocate the affectionate behavior to nurture a bond in the grandparent-grandchild (GP-GC) relationship [41, 7, 8].

Several studies have shown that there are four main factors that conceptualize grandchildren received affection, which include love and esteem, caring, memories and humor, and celebratory affection [7, 12]. Love and esteem refer to “expressions of love, compliments, relationship importance, and recipient self-worth” [12] (p. 81). Caring refers to an expression of concern toward a grandchild’s life and serving as an attentive listener [12] (p. 80). Memories and humor refers to the sharing of stories, and this includes “jokes and humor” [12] (p. 81). Lastly, celebratory affection refers to “acknowledging special occasions” that are important to a grandchild’s life (i.e., sending a birthday card) [12] (p. 81). Thus, these four factors have conceptualized grandchildren received affection from their grandparents.

This study’s purpose is to use AET framework to explore whether grandmother affection is linked to their adult grandchildren’s practice of health behaviors including dietary behaviors, exercise behaviors, substance abuse behaviors, and sleep habit behaviors. Given that AET framework suggests that people maintain relational bonds to promote *survival* and *procreation* [38, 39], it is important to examine whether grandchildren received affection is linked to grandchildren’s health behaviors, which may prolong their overall health and well-being [42].

### Affection and grandchildren’s health behaviors

While previous studies have highlighted the correlation between health outcomes and expressed affection [43–45], this study seeks to examine the health behavior outcomes of received affection in GP-GC relationships. Most of the literature has provided significant attention to expressed affection, but there is still a need to further investigate the role of received affection to grandchildren’s health outcomes [46]. Previous research has examined received affection in other family relational contexts. For example, Schrodt al. (2007) found that received parental affection mediated the relationship between open conversation patterns and adult children’s self-esteem and mental health [47]. More recently, Floyd (2014), found that being deprived from receiving affection is linked to mental health issues (i.e., loneliness, depression). The main study that has examined received affection and health outcomes in GP-GC relationships has been conducted by Mansson (2013c), which found that young adult grandchildren’s mental well-being (e.g., stress, depression, loneliness) was negatively correlated with certain types of received affection (e.g., memories and humor, celebratory). However, Mansson (2013c) did not find a link between grandchildren’s mental well-being and love and esteem, or caring affection. Thus, this study seeks to extend the work of health behavioral outcomes and received affection by investigating behavioral health outcomes and received affection in GP-GC relationships. To our knowledge, there has not been a study that has examined the link between received affection and health behavioral outcomes of grandchildren. Because studies have emphasized relationship characteristics such as the relational closeness and intimacy in the grandmother-grandchild relationship, there is a gap in not knowing the health outcomes of received grandmother affection. Researchers that have looked at health outcomes have focused on grandparents’ health outcomes, such as Kelley et al. (2020) and Tang et al. (2016) who found that grandparents who raise grandchildren who achieve relational closeness gain positive mental health and well-being benefits [48, 49]. The present study seeks to extend prior work by examining grandmothers’ received affection in the grandmother-grandchild relationship, and investigating the positive links to grandchildren health outcomes.

Previous research has examined the role of emotional and social support to behavioral outcomes in a variety of relational contexts. For instance, receiving social support from a peer has shown to influence young adult’s diet, exercise, sleep, and substance abuse [50, 51]. Peer support has also been shown to increase the odds of promoting healthy behaviors such as making healthier diet decisions, sleeping adequately, exercising regularly, and avoiding substance use [51, 52]. In another study that examined the role of grandparents and parents, it was found that grandparents and parent caregivers who withheld affection and used harsh discipline methods such as spanking and screaming and poor monitoring led to increased substance abuse risks such as binge drinking, marijuana use, and cigarette use [53]. Additionally, O’Leary and Butler (2015) found evidence that grandparents provide social and financial support by funding grandchildren’s drug rehabilitation treatments to aid the reduction of substance abuse behavior [54]. Another study found that when parents engage in substance abuse behavior, grandparents become more involved in the care of their grandchildren, and grandparents take the responsibility to talk about issues related to substance abuse and also provided emotional support to their grandchildren to prevent grandchildren from engaging in future substance abuse [55]. Moreover, it has been found that isolated young adults who do not feel emotionally cared for, or supported tend to be at a higher risk for alcohol consumption, tobacco use, and drug use [56]. Other studies have also found that those without emotional bonds and support systems risk adopting unhealthy patterns such as making poor diet choices (e.g., donuts, soda), lack of physical activity, inadequate sleep, and substance abuse [50, 57–59]. Because grandmothers are more likely to express higher levels of affection to their grandchildren than grandfathers given their gender socialization of being expected to be nurturing and caring toward their grandchildren, this study will exclude grandfathers [60]. Prior studies have established preliminary evidence of the caregiving role of grandparents on grandchildren’s health behaviors; however, this study will extend prior work by examining whether young adult grandchildren’s health behaviors (i.e., diet, sleep, substance abuse) are influenced by the received affection from their grandmothers.

Several studies have investigated grandchildren’s healthy eating patterns and exercise behaviors, and they find that caregiver figures, such as grandparents are influential in the health decision making process. For instance, Farrow (2014) found that grandparents provided healthier meals and role modeled healthy food intake behavior to their grandchildren in comparison to parents, and grandparents were able to provide grandchildren with healthy food to help their grandchildren engage in emotional regulation [61]. Another study found that grandparents provided breakfast and lunch to their younger grandchildren and dinner for older grandchildren including fresh fruit, vegetables, milk, grain and cereal foods, and meat [62]. In terms of physical engagement, Viguer et al. (2010) found that grandchildren engage in physical activities with their grandparents such as going for a walk, going to the park, going camping, and going to the beach [63]. In terms of sleep behaviors, Li et al. (2021) found that grandparents can have a higher influence on their grandchildren’s sleep behaviors such as resisting bedtime in comparison to their parents [64]. Further, another study found that grandmothers tend to have an influence on children who are at a high risk for substance abuse disorders [65]. A family’s history of substance abuse (i.e., alcohol) also can affect college students’ alcohol consumption [66]. These studies suggest that the relationships in the family context may influence young adult’s health behaviors.

Affection exchange theory provides an opportunity to investigate whether received affection is linked to health behavioral outcomes (i.e., diet, exercise, substance abuse, sleep). As discussed previously, AET discusses the main role for promoting *survival* and *procreation* [38, 39]. If grandchildren receive affection from their grandmothers, they may be more likely to make wiser health behavioral decisions, which can help them live longer and attract higher quality mates in the mate selection process [67, 38, 50]. Thus, if grandchildren eat healthier, exercise regularly, sleep adequately, and avoid substance abuse (e.g., alcohol, drugs), then they may be more likely to remain genetically fit [67]. As we know, affection provides positive benefits to individuals’ emotional well-being, but it is not yet known whether this is the case for their health behavioral patterns. Thus, the objective of this study is to examine the health outcomes of received affection from grandmothers in relation to grandchildren’s health behaviors (i.e., dietary behaviors, exercise behaviors, substance abuse behaviors, and sleep habit behaviors). Based on AET’s predictions and the previous literature findings that connect received affection with health behavioral outcomes, the following set of hypotheses are posed to investigate whether received affection from grandmothers is positively connected to young adult grandchildren’s reports of health behaviors.

- **H1:** Received affection from grandmothers (i.e., love and esteem, caring, memories and humor, and celebratory) is positively related to grandchildren’s reports of their dietary behaviors.
- **H2:** Received affection from grandmothers (i.e., love and esteem, caring, memories and humor, and celebratory) is positively related to grandchildren’s reports of their exercise behaviors.
- **H3:** Received affection from grandmothers (i.e., love and esteem, caring, memories and humor, and celebratory) is inversely related to grandchildren’s reports of their substance abuse behaviors.
- **H4:** Received affection from grandmothers (i.e., love and esteem, caring, memories and humor, and celebratory) is positively related to grandchildren’s reports of their sleep habit behaviors.

## Method

### Participants

This study used a cross-sectional approach with a convenience sampling design. The sample included (67.2% women and 32.8% men) 229 students in a large Western university in the United States. The average age of the participants was 19 (*SD* = 3.05; range = 18-40). The ethnic background of participants was composed of 18.8% European-American, 6.1% African-American, 2.6% Native-American, 16.6% Asian-American, 44.5% Hispanic-American, and 11.4% Other. The highest education level of education indicated by the participants was 30.6% high school graduate, 63.3% some college, 3.1% college graduate, .4% some high school/GED, and 2.6% post graduate. The highest level of education that their grandmother achieved was 54% some high school, 24.4% high school graduate, 2.8% technical training, 7.5% some college, 8.5% college graduate, and 2.8% post graduate. The nature of their perceived relationship identified was 82.5% biological and 17.5% non-biological.

### Procedure

This study was approved by the Institutional Review Board of a mid-sized, western public university in the United States (#110513). Participants were recruited in communication courses from the College of Fine Arts and Humanities. Faculty distributed the recruitment letter of this study in their courses. Participants were invited to participate in an online grandparent survey in exchange of a small amount of extra credit (2% of total course points). The inclusion criterion was that participants had to be at least 18 years of age and have had an existing biological or non-biological grandmother-grandchild relationship that was perceived to be close. To comply with the IRB’s ethical guidelines, participants completed a virtual consent form prior to completing the questionnaire. The timing of the sampling was during the fall months of October 15 to December 15. Participants that provided consent to participate were referred to a web link via Qualtrics to complete a 15 min questionnaire. In addition, participants were asked to respond to questions about their demographics, their received grandmothers’ affectionate communication, and their health behaviors.

### Measures

#### Grandchildren’s Received Affection

The Grandchildren Received Affection Scale [19] was used to assess grandchildren’s perceptions of their grandmother’s affection using a 17-item, 7-point Likert Scale. Mansson (2013) validated this scale with effective reliability using Cronbach’s alpha values [19]. The initial reliabilities of the subscales included love and esteem (α = .91, *n* = 5), caring (α = .91, *n* = 5), memories and humor (α = .78, *n* = 4), and celebratory affection (α = .73, *n* = 3) [19]. In the present study, SPSS was used to calculate Cronbach’s alpha reliability values. The summation subscales and the alpha reliabilities of the subscales in this study include love and esteem (α = .96), caring (α = .96), memories and humor (α = .94), and celebratory affection (α = .85), which suggest a high reliability across subscales. Participants responded to items such as whether their grandmother told them that she loved them, missed them, asked them how they were doing, asked about their life, and told them stories about her life. Items ranged from 1 (*strongly disagree*) to 7 (*strongly agree*), with higher values indicating higher degree of received affection. Specific items used in this study are listed in the Additional file 1.

#### Health behaviors

Health behaviors were examined using a 53-item Health Practices Scale [50], which is a 7-point Likert scale (e.g., 1 = almost never; 7 = almost always) based on based on the frequency of health behaviors. This scale was validated by Jackson (2006) and the internal reliability of the subscales included diet (α = .92), exercise (α = .92), substance abuse (α = .89), and sleep (α = .89). In the present study, subscales included diet, exercise, substance abuse, and sleep to assess grandchildren’s overall health behaviors (See Additional file 1).

#### Diet

A 21-subscale in the Health Practices Scale was used to assess eating behaviors. Sample items included, “limit amount of fat in diet,” “limit sugar intake,” and “eat healthy foods” [50]. Participants indicated the frequency level of how often they performed a set of dietary behaviors, from 1 (*almost never*) to 7 (*almost always*). The alpha reliability of this scale was .92 using SPSS’s Cronbach’s alpha reliability test, which indicated high internal reliability.

#### Exercise

An 11-item subscale in the Health Practices Scale was used to assess exercise behaviors. Sample items included, “do exercises that are good for you,” “go for regular walks,” and “make sure you are physically active” [50]. Participants indicated the frequency level of how often they performed a set of exercise behaviors, from 1 (*almost never*) to 7 (*almost always*). The alpha reliability of this scale was .94 using SPSS’s Cronbach’s alpha reliability test, which indicated high internal reliability.

#### Substance abuse

An 11-item subscale in the Health Practices Scale was used to assess substance abuse behaviors [50]. Sample items included, “smoke cigarettes daily,” “use recreational drugs to relax,” and “drink alcohol until intoxicated.” Participants indicated the frequency level of how often they performed a set of substance abuse behaviors, from 1 (*almost never*) to 7 (*almost always*). The alpha reliability of this scale was .87 using SPSS’s Cronbach’s alpha reliability test, which indicated high internal reliability.

#### Sleep

A 4-item subscale in the Health Practices Scale was used to assess individual’s sleep behaviors [50]. Sample items included, “sleep 7-8 hours per night” and “get adequate sleep every day.” Participants indicated the frequency level of how often they performed a set of substance sleep behaviors, from 1 (*almost never*) to 7 (*almost always*). The alpha reliability of this scale was .80 using SPSS’s Cronbach’s alpha reliability test, which indicated high internal reliability.

### Preliminary analysis

A correlation analysis was performed among the variables of this study to obtain the Pearson Product moment correlation coefficients of this study. Correlations for all of the variables were examined to determine strong relationships and high internal reliability across instruments used in this study (see Table 1).

**Table 1:** Reporting Zero-Ordered Means, Standard Deviations, and Correlation Matrix

Measure	1	2	3	4	5	6	7	8
1. Love and esteem	1							
2. Caring	0.83**	1						
3. Memories and humor	0.72**	0.67**	1					
4. Celebratory	0.55**	0.55**	0.43**	1				
5. Diet	0.09	0.14*	0.18*	0.21**	1			
6. Exercise	0.15*	0.22**	0.25**	0.23**	0.52**	1		
7. Substance Abuse	0.01	−0.04	−0.05	0.09	0.01	0.08	1	
8. Sleep	0.22**	0.14*	0.21**	0.14*	0.21**	0.07	0.01	1
α	0.96	0.96	0.94	0.85	0.92	0.94	0.87	0.80
M	9.92	10.22	7.71	5.67	23.74	14.03	5.55	4.80
SD	1.66	1.48	1.36	1.04	6.59	4.50	3.30	1.69

*Note*. *p < .05. ** *p* < .01

The following assumptions were checked to ensure that the regression assumptions were met. First, there was evidence of a linear relationship between the independent variables (received affection) and health outcome variables. Second, the residuals were normally distributed, which demonstrated the normality assumption. Third, the scatterplots were spread for best line fit, which fulfilled the homoscedasticity assumption. Fourth, the observations were independent.

Fifth, this study tested for multicollinearity. The tolerance statistics (TS) and variance inflation factor (VIF) were examined in the regression analyses to uncover any possible multicollinearity issues among the independent variables. The variance inflation factor (VIF) was used to determine if the variance of the estimated regression coefficient was inflated due to collinearity among the independent variables or predictors of the regression models. The lowest tolerance statistic was .25 for received grandmother affection (love and esteem) and the highest VIF was 1.11 for received grandmother affection (celebratory), which showed that multicollinearity was not a main concern in this study given Mertler and Vannatta’s (2002) recommendations that VIF values should be under 2.5 [68]. The tolerance statistic was obtained using IBM SPSS 23.0, which regressed independent variables in the regression equations (received affection variables). The following equation was used to calculate the VIF of the variance of each of the regression models.

$$VIF=\frac{1}{1-{R}_i^2}$$

The VIF values of the four regression models including each of the independent variables with each health outcome included: dietary behaviors (VIF = 1.08, R^2^ = .07), exercise behaviors (VIF = 1.11, R^2^ = .10), substance abuse (VIF = 1.02, R^2^ = .02), and sleep behaviors (VIF = 1.06, R^2^ = .06).

Four multiple regression models are specified in the analysis of this study. Each of the models has the following independent variables entered into the regression block in each of the regression equations: love and esteem (X_1_), caring (X_2_), memories and humor (X_3_), and celebratory affection (X_4_). The βo is the intercept and the βi is the slope for each independent variable. The first model testing the first hypothesis will examine received grandmother affection on diet behavior (Y).

$$Diet\ (Y)={\beta}_o+{\beta}_ix1+{\beta}_ix2+{\beta}_ix3+{\beta}_ix4$$

The second model testing the second hypothesis will examine received grandmother affection on exercise behavior (Y).

$$Exercise\ (Y)={\beta}_o+{\beta}_ix1+{\beta}_ix2+{\beta}_ix3+{\beta}_ix4.$$

The third model testing the third hypothesis will examine received grandmother affection on diet behavior (Y).

$$Substance\ Abuse\ (Y)={\beta}_o+{\beta}_ix1+{\beta}_ix2+{\beta}_ix3+{\beta}_ix4$$

The fourth model testing the last hypothesis will examine received grandmother affection on diet behavior (Y).

$$Sleep\ Habits\ (Y)={\beta}_o+{\beta}_ix1+{\beta}_ix2+{\beta}_ix3+{\beta}_ix4$$

The decision criteria for the correlation, coefficient of determination, and regression slope of significance in this study included: *p* < .05. If the *p*-value was below .05, this was an indicator of statistical significance. However, if the *p*-value was above this criteria, this would indicate nonsignificant findings. In the regression models, the *F* statistic value was also used as a measure of overall fit, which indicated the probability that the models cannot be rejected. Additionally, the mean squared error values were also used to support the regression line assumption. Lastly, the estimation of statistical uncertainty was captured by the standard error of the regression model parameters using residual plots and regression diagnostics, which indicated that the regression models were reasonable.

### Statistical analysis

All statistical analyses were executed using IBM SPSS 23.0. Hierarchical multiple regression models were used to assess the four hypotheses in this study. The first regression model examined whether received affection from grandmothers was related to grandchildren’s dietary behaviors (H1). The second regression model examined whether received affection from grandmothers was related to grandchildren’s exercise behaviors (H2). The third regression model examined whether received affection from grandmothers was related to grandchildren’s substance abuse behaviors (H3). And, the final regression model examined whether received affection from grandmothers was related to grandchildren’s sleep habit behaviors (H4).

## Results

Summaries of the multiple regression findings are in Table 2. Hypothesis one posed that received affection (i.e., love and esteem, caring, memories and humor, and celebratory) from grandmothers would be positively related to grandchildren’s reports of their dietary behaviors. The results of a multiple regression analysis revealed a significant model [*R*^2^ = .07, *F*(4, 200) = 3.77, *p* < .01]. Grandchildren’s reports of received memories and humor (β = .20, *t* = 2.01, *p* < .05, *pr*^2^ = .14), and celebratory (β = .20, *t* = 2.49, *p* < .05, *pr*^2^ = .17) affection from their grandparents was associated to grandchildren’s reports of their dietary behaviors. However, grandchildren’s reports of received love and esteem (β = −.24, *t* = −1.80, *p* = .06, *ns*), and caring (β = .11, *t* = .84, *p* = .09, *ns*) was not associated to grandchildren’s reports of their dietary behaviors. Therefore, this hypothesis was partially supported.

**Table 2:** Results of multiple regression analyses between the affection variables and health behaviors

		Diet			Exercise			Substance Abuse			Sleep	
	*t*	β	*pr*^2^	*t*	β	*pr*^2^	*t*	β	*pr*^2^	*t*	β	*pr*^2^
1. Love and esteem	−1.80	−0.24	−0.13	−2.04	**−**0.26*	−0.14	0.80	0.11	0.05	2.01	0.26*	0.14
2. Caring	0.84	0.11	0.06	1.35	0.17	0.09	−1.18	−0.15	−0.08	−1.58	−0.19	−0.11
3. Memories and humor	2.01	0.20*	0.14	2.66	.25**	0.18	−0.87	−0.09	−0.06	1.32	0.13	0.09
4. Celebratory	2.49	0.20*	0.17	2.26	.18*	0.15	1.84	0.15	0.13	0.53	0.04	0.04

*Note*. *p < .05. ** *p* < .01. β = standardized beta coefficients

Hypotheses two predicted that received affection (i.e., love and esteem, caring, memories and humor, and celebratory) from grandmothers would be positively related to grandchildren’s reports of their exercise behaviors. The results of a multiple regression analysis revealed a significant model [*R*^2^ = .10, *F*(4, 212) = 5.92, *p* < .001]. Grandchildren’s reports of received love and esteem (β = −.26, *t* = −2.04, *p* < .05, *pr*^2^ = −.14), memories and humor (β = .25, *t* = 2.65, *p* < .01, *pr*^2^ = .18), and celebratory (β = .18, *t* = 2.26, *p* < .05, *pr*^2^ = .15) affection from their grandmothers was associated to grandchildren’s exercise behaviors. However, grandchildren’s reports of received caring (β = .17, *t* = 1.35, *p* = .08, *pr*^2^ = .09, *ns*) affection from their grandmothers was not associated to grandchildren’s exercise behaviors. Thus, this hypothesis was partially supported.

Hypotheses three predicted that received affection (i.e., love and esteem, caring, memories and humor, and celebratory) from grandmothers would be inversely related to grandchildren’s reports of their substance abuse behaviors. The results of a multiple regression analysis revealed a nonsignificant [*R*^2^ = .02, *F*(4, 213) = 1.29, *p* > .05, *ns*]. Grandchildren’s reports of received love and esteem (*p* = .08, *ns*), caring (*p* = .10, *ns*), memories and humor (*p* = .14, *ns*), and celebratory (*p* = .23, *ns*) affection from their grandmothers was not associated to grandchildren’s substance abuse behaviors. Thus, this hypothesis was not supported.

Hypothesis four predicted that received affection (i.e., love and esteem, caring, memories and humor, and celebratory) from grandmothers would be positively related to grandchildren’s reports of their sleep habit behaviors. The results of a multiple regression analysis revealed a significant model [*R*^2^ = .06, *F*(4, 218) = 3.75, *p* < .01]. Grandchildren’s reports of received love and esteem (β = .26, *t* = 3.43, *p* < .05, *pr*^2^ = .14) affection from their grandmothers was associated to grandchildren’s sleep habit behaviors. However, grandchildren’s reports of received caring (β = −.19, *t* = −1.58, *p* = .12, *ns*), memories and humor (β = .13, *t* = 1.32, *p* = .20, *ns*), and celebratory (β = .04, *t* = .53, *p* = .52, *ns*) affection from their grandmothers was not related to grandchildren’s sleep habit behaviors. Thus, this hypothesis was partially supported.

## Discussion

Affection exchange theory (AET) provided a framework to be able to address this study’s aim of explaining the link between adult grandchildren’s reports of their grandmothers’ affection on their individual health. This study contributes to prior research by examining AET using grandchildren’s received affection along with their health perceptions. In this study, the first hypothesis was partially supported because memories and humor and celebratory affection related with grandchildren’s dietary behaviors; however, love and esteem, and caring were not related with these behaviors. The second hypothesis was partially supported because love and esteem, memories and humor, and celebratory affection related to grandchildren’s exercise behaviors; however received caring was not shown to be related. The third hypothesis indicated that received affection did not relate with grandchildren’s substance abuse behaviors. The last hypothesis was partially supported because love and esteem related to grandchildren’s sleep habit behaviors, but other affection factors did not relate to this behavior.

Receiving grandmothers’ affection was partially linked to grandchildren’s reports of their dietary behaviors. More specifically, grandchildren’s reports of received memories and humor, and celebratory affection from grandparents was a predictor for dietary behaviors. Affection Exchange Theory postulated that the expression of affection can serve to promote genetic fitness [39]. By college students establishing healthy dietary behaviors, they in turn, advance their overall well-being, and their likelihood of procreation and longevity [69]. Mansson’s (2013) study demonstrated that grandparent affection did correlate with college students’ mental health, which can promote their healthy decision-making process [7]. Further, Floyd (2014) demonstrated that the lack of affection has been correlated with negative health outcomes (i.e., stress, depression) [10]. Studies by Weisbuch et al. (2011) and Stroberg and Humphrey (1987) have also shown that the emotional bonds established in relationships can impact abnormal eating behavior patterns in adults [70, 71]. Past studies have found that grandparents play a role in the healthy food intake of grandchildren including fruit and vegetables [61, 62]. The dietary intake of grandchildren and their food choices during early grandparent caregiving years may influence young adult grandchildren’s dietary habits [72]. A study found that grandparents prioritize feeding their grandchildren healthy foods and treats, which set the development of healthy eating behavioral patterns and food preferences, which may influence college students’ future dietary choices [73]. College students who interact in social relationships such as family relationships have also been showed to be related to sustaining a healthy diet [74]. However, the present study differs from these prior studies by examining the grandmother-grandchild relationship in relation to healthy dietary behaviors among college students. In particular, the present study extends prior work by demonstrating a link between grandchildren’s received grandmother affection and their grandchildren’s healthy dietary behaviors. However, there is still a need to further explore this finding longitudinally in future studies.

This study also showed that grandchildren’s received love and esteem, and caring were not predictors of college students’ dietary behaviors. One reason for this finding might be because positive affect alone might not include discussions about college students’ dietary needs. If a college student feels loved and cared for, this factor alone may not carry enough predictor weight to influence grandchildren’s healthy dietary behaviors. Perhaps there are other factors that come into play such as college stress and the mental health, which may also affect college students’ dietary behaviors [75]. Future studies may need to continue to examine the correlations between affection and college students’ dietary habits to gain further insight into this study’s findings.

Grandchildren’s reports of received love and esteem, memories and humor, and celebratory affection from grandmothers were predictors of grandchildren’s exercise behaviors. Affection exchange theory would suggest that affection can serve as a drive to maintain the emotional bond in the grandparent-grandchild relationship, which can promote the genetic fitness across generations [19, 39]. Prior cross-sectional family studies have investigated the link between family members (i.e., grandparents, parents) emotional and social support, and the physical activity of adolescents [76–78]. For instance, Xie et al. (2018) found that direct social support from Latino grandparents led to doing physical activities with grandchildren, asking grandchildren to do physical activities, and rewarding grandchildren for doing physical activities [76]. Intergenerational relationships such as the grandparent-grandchild relationship are also shown to be strengthened by the physical activity influence of grandparents on their young adult grandchildren [77]. In a parent and peer context, Haidar et al. (2019) found that receiving parental and peer social support was positively associated with healthier physical activity among adolescents [78]. However, what was missing from these studies was the investigation of the affectionate aspect in the grandmother-grandchild relationship in relation to grandchildren’s exercise behavior. This study extends prior work by finding that received affection from a grandmother may positively, yet indirectly influence college students’ exercise habits. Interestingly, grandchildren’s reports of received caring affection not associated with grandchildren’s exercise behaviors. This might be because the demonstration for caring may not depend on whether a grandchild exercises, or not. More research needs to explore the role of affection in mediating grandchildren’s’ exercise behaviors, to determine the role grandmothers play in the physical activity process of young adults.

Interestingly, substance abuse behaviors was not related to grandmothers’ affection toward their grandchildren. Lange and Greif (2011) found that grandmothers are known to provide care for children with mothers with substance use disorders, and these adolescents are at a high-risk for engaging in substance abuse as they reach adulthood [65]. However, the present study did not find any link between the expression of grandmothers’ affection and their grandchildren’s substance abuse. One reason may be that affection cannot necessarily predict substance abuse behavior that can occur due to other factors. For instance, previous studies have shown that family’s history (i.e., parents) of substance abuse is correlated with young adult’s substance use [79, 80]. On the other hand, Caday (2017) found that the lacked of received affection and attention from family members among young adults are some of the reasons why young adults become dependent on substance abuse during their college years [81]. Thus, even though this study did not find a link between grandmothers’ affection and their grandchildren’s’ substance abuse behavior, this is still a worthy area for further investigation in family communication.

This study also found that grandmother’s expression of love and esteem affection was associated with their grandchildren’s sleep habit behaviors. This supports a study that suggests that emotional bonding in relationships can affect sleep as a health related behavior [82]. It has previously been shown that social support and integration can be correlated with undergraduate college student’s sleep status [83]. Grandchildren who both receive affection and sleep adequately, may be at a higher advantage in their overall school performance as well [84]. Those that suffering sleep problems and who lack emotional connection with a loved family member (i.e., grandmother), can suffer from stress and pressure [85].

On the other hand, grandparents’ caring, memories and humor, and celebratory affection was not related to grandchildren’s sleep habit behaviors. This suggests that different types of affection will not necessarily make an impact on whether young adult grandchildren will obtain better quality sleep at night. This might be due to the convenience sample that was used was college students, which are commonly known to suffer from lack of sleep due sleep disturbances such as and other academic pressures [86]. Another reason might be that affection might not be a strong predictor of sleep quality in the grandparent-grandchild relationship context. However, perhaps exploring affection in other relationship contexts such as romantic relationship, this correlation may be more apparent.

### Strengths

The current study presented strengths that should be discussed. First, this study used the affection exchange theory to discover the link between adult grandchildren’s reports of received grandmother affection and their own report of health behavior. To date, there has been little research conducted to find the link in perceived affection from grandparents to specific health behaviors such as diet, exercise, no substance abuse, and sleep habit behaviors. Second, the sample demonstrated ethnic diversity, which may be representative to a diverse population. Third, the correlational and regression methodology employed was effective in testing the hypotheses using a moderate sample size.

### Limitations and future directions

While the study had several strengths, this study also had several limitations that will be discussed along with ideas for future directions. First, this study relied on self-report surveys that were conducted online via the use of Qualtrics and it asked students to recall their grandmothers’ affectionate communication behavior. While self-report surveys are generally acceptable to use, there are other possible methods that could be explored to overcome the limitations of recalling information or the use of deception in self-report surveys. For example, future studies can use a variety of different methods to explore different variables to measure affection differently. For instance, future observational studies may audio record and/or video record grandmothers providing affection to their grandchildren and then have grandchildren assess whether grandchildren actually perceive this behavior as affectionate communicative behavior. Second, this study only relied on the perceptions of the grandchildren. A future study may continue this line of research using dyadic data analysis between grandchildren and their grandmothers, and grandchildren’s behavioral health factors. Third, this study focused only on grandmothers. Future studies can focus on grandfathers and their grandchildren to determine if grandfathers’ affection will correlate with their grandchildren’s health behaviors. Fourth, this study used a convenient sample of college students who had an existing grandmother-grandchild relationship given the study’s focus on college-aged grandchildren. However, future studies can use different sampling designs such as systematic sampling using a larger sample to study different aged grandchildren to determine if grandmothers’ affection affect their grandchildren in elementary, middle school, or high school. Lastly, this study did not examine additional relational factors such as the length and quality of the affectionate relationship. Future researchers may examine how these relational factors influence grandchildren’s health outcomes.

## Conclusion

To conclude, the aim of this study was to examine the influence of received grandmother affection on their grandchildren’s health behaviors such as their a) diet, b) exercise, c) substance abuse, and d) sleep. In terms of health outcomes, this study showed that received memories and humor, and celebratory affection was influential to grandchildren’s reports of dietary behaviors. Also, received love and esteem, memories and humor, and celebratory affection was associated with grandchildren’s exercise behaviors. However, none of the grandparent affection factors were found to have relationship with grandchildren’s substance abuse behaviors. In terms, of sleep, only love and esteem impacted grandchildren’s sleep habit behaviors. Thus, this study overall highlights the importance of examining affection exchange theory’s variables for affection and the predictors in terms of promoting emotional bonds among grandmothers and their children, and this study demonstrated that receiving affection from grandmothers may partially impact the health outcomes of young adult grandchildren.

## Supplementary Information

**Additional file 1.**

## Data Availability

The data set used in this study is available from the corresponding author on a reasonable request at lsalazar@wtamu.edu.
