# Supplementary material for: The effects of received grandmothers’ affection on adult grandchildren’s health behaviors using affection exchange theory
Source: BMC Public Health. 2022 Apr 11;22:714. doi: 10.1186/s12889-022-13049-4 (PMC9004156; doi:10.1186/s12889-022-13049-4)
Supplement: Supplementary file 1 — Additional file 1. [file 12889_2022_13049_MOESM1_ESM.docx]

| 1. Tells me she loves me. | 11. Tells me stories about her life. |
| --- | --- |
| 2. Tells me that she misses me. | 12. Tells me jokes. |
| 3. Tells me she is proud of me. | 13. Tells me about fun memories from her past. |
| 4. Tells me she enjoys spending time with me. | 14. Tells me funny stories. |
| 5. Tells me I’m special to her. | 15. Gives me money. |
| 6. Asks me how things are going. | 16. Send cards for my birthday and holidays. |
| 7. Asks how I am doing. | 17. Gives me gifts on special occasions. |
| 8. Listens to what I have to say. |  |
| 9. Pays attention to me when I talk. |  |

ADDITIONAL FILE 1

Grandchildren Received Affection Scale

My grandmother…

**Reference:**

Mansson DH. The grandchildren received affection scale: Examining affectual solidarity factors. South Commun J. 2013; 78(1):70-90. https://doi.org/10.1080/1041794X.2012.729124

**Notes:**

17-item scale

Alpha reliabilities:

Love and esteem = alpha .91 (n=5); Caring = alpha .91 (n=5); Memories and humor = .78 (n = 4); Celebratory = alpha .73 (n=3)

Health Practice Items

*Diet*

| 1. Limit amount of fat in diet | 12. Drink eight or more glasses of water per day |
| --- | --- |
| 2. Limit amount in red meat in diet | 13. Keep track of the number of calories in foods you are eating |
| 3. Eat non-fat dairy products | 14. Figure out from labels what foods are good for you |
| 4. Limit sugar intake | 15. Eat junk food * |
| 5. Eat four servings of vegetables per day | 16. Eat whole grain foods |
| 6. Eat healthy foods | 17. Eat food high in fiber |
| 7. Limit salt intake | 18. Consume enough calcium |
| 8. Eat a balanced diet | 19. Read food labels |
| 9. Consume enough vitamins and minerals | 20. Ignore the total calories in your food * |
| 10. Limit the amount of sweets in your diet | 21. Consume fatty foods * |
| 11. Eat fast food * |  |

*Exercise*

| 22. Exercise vigorously | 28. Exercise so you are breathing heavily |
| --- | --- |
| 23. Perform stretching exercises | 29. Avoid exercising * |
| 24. Have a physically active home life | 30. Get daily aerobic exercise |
| 25. Do exercises that are good for you | 31. Make sure you are physically active |
| 26. Go for regular walks | 32. Walk or run for a mile or longer at least three times per week |
| 27. Do physical exercises you enjoy |  |

*Substance Abuse*

| 33. Have five or more alcoholic drinks per day | 39. use recreational drugs to relax |
| --- | --- |
| 34. Use drugs to get high | 40. Have contact with cigarette smoke |
| 35. Avoid getting second-hand smoke * | 41. Smoke excessively |
| 36. Avoid using tobacco products * | 42. Drink alcohol until intoxicated |
| 37. Limit your intake of alcohol * | 43. Drink alcohol excessively |
| 38. Smoke cigarettes daily |  |

*Sleep*

| 44. Sleep 7-8 hours per night |
| --- |
| 45. Get adequate enough sleep and rest |
| 46. Not get enough sleep and rest |
| 47. Feel rested and refreshed |

**Reference:**

Jackson T. Relationships between perceived close social support and health practices within community samples of American women and men. J Psychol. 2006;140(3):229-246. https://doi.org/10.3200/JRLP.140.3.229-246

**Notes:**

7-point Likert scale (47-item scale)

1 (never) to 7 (always)

Alpha reliabilities:

Substance abuse = alpha .89; Sleep = alpha .89; Regular exercise = .92; Healthy diet = .92; Diet (n = 21)

Exercise (n = 11); Substance Abuse (n = 11); Sleep (n = 4)
